# Circulating concentrations of endothelin-1 predict coronary heart disease in women but not in men: a longitudinal observational study in the Vara-Skövde Cohort

**DOI:** 10.1186/s12872-015-0141-y

**Published:** 2015-11-14

**Authors:** Bledar Daka, Josefin Olausson, Charlotte A. Larsson, Margareta I. Hellgren, Lennart Råstam, Per-Anders Jansson, Ulf Lindblad

**Affiliations:** Department of Public Health and Community Medicine, Institute of Medicine, Sahlgrenska Academy, University of Gothenburg, Box 454, S-405 30 Gothenburg, Sweden; Department of Molecular and Clinical Medicine, The Wallenberg Laboratory, Institute of Medicine, Sahlgrenska Academy, University of Gothenburg, Gothenburg, Sweden; Department of Clinical Sciences, Social Medicine and Global Health, Lund University, Malmö, Sweden; Department of Clinical Sciences, Family Medicine, Lund University, Malmö, Sweden

**Keywords:** Coronary heart disease, Endothelin-1, Prospective study, Skaraborg project

## Abstract

**Background:**

The vasoconstricting peptide endothelin-1 has been proposed to be a marker of cardiovascular disease. Our aim was to investigate whether circulating endothelin-1 levels predict coronary heart disease (CHD) in Sweden.

**Methods:**

In 2002–2005, 2816 adult participants (30–74 years) were randomly selected from two municipalities in south-western Sweden. Cardiovascular risk factors and endothelin-1 levels were assessed at baseline, and incident CHD was followed-up in all participants through 2011. After exclusion of 50 participants due to known CHD at baseline and 21 participants because of unsuccessful analysis of endothelin-1, 2745 participants were included in the study. In total, 72 CHD events (52 in men and 20 in women) were registered during the follow-up time.

**Results:**

We showed that baseline circulating endothelin-1 levels were higher in women with incident CHD than in women without CHD (3.2 pg/ml, SE: 0.36 vs 2.4 pg/ml, SE: 0.03, *p* = 0.003) whereas this difference was not observed in men (2.3 pg/ml, SE: 0.16 vs 2.3 pg/ml, SE: 0.04, *p* = 0.828). An age-adjusted Cox proportional regression analysis showed an enhanced risk of CHD with increasing baseline endothelin-1 levels in women (hazard ratio (HR) = 1.51, 95 % CI = 1.1–2.1, p = 0.015) but not in men (HR = 0.98, 95 % CI = 0.8–1.2, *p* = 0.854). Furthermore, the predictive value of endothelin-1 for incident CHD in women was still significant after adjustments for age, HOMA-IR, apolipoprotein (apo)B/apoA1 and smoking (HR = 1.53, CI = 1.1–1.2, *p* = 0.024).

**Conclusion:**

Circulating endothelin-1 levels may predict CHD in women.

## Background

Coronary heart disease (CHD) refers to a wide spectrum of illnesses including coronary atherosclerosis, myocardial infarction and post-infarct heart failure [1]. CHD is one of the leading causes of mortality among men and women causing almost 1.8 million deaths per year in Europe [2]. Endothelin-1, which is elevated in endothelial dysfunction, has been proposed to be a marker of cardiovascular disease [3]. Thus, measurements of endothelin-1 might offer insight into subclinical stages of CHD and guide clinicians when to initiate primary and secondary prevention [4].

Circulating endothelin-1 levels have been shown to increase in patients with advanced atherosclerosis and coronary artery disease progression [5, 6]. Moreover, earlier studies adopting a cross-sectional or retrospective study design demonstrated that circulating endothelin-1 levels were associated with CHD in women of all ages while this association was only observed in older men, thus, suggesting a predictive role of circulating endothelin-1 for future CHD primarily in women [6–8]. A recent prognostic observational study in selected patients showed a predictive role of circulating endothelin-1 levels on the first manifestations of atherosclerotic disease [9]. Here, we performed a longitudinal population-based study of the Vara-Skövde cohort [10] to test the hypothesis that circulating endothelin-1 levels are predictive of incident CHD events.

## Methods

### Study population

**Fig 1:**
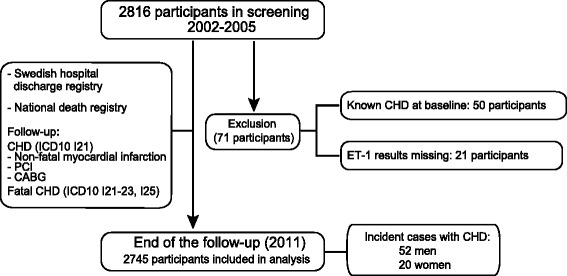
Schematic overview of study design and follow-up of the study. CHD; coronary heart disease, ET-1; endothelin-1, PCI; percutaneous coronary intervention, CABG; coronary artery bypass grafting

The Vara-Skövde cohort has been previously described [10]. Briefly, between 2002 and 2005 a cohort based on a random age- and sex-stratified sample of subjects aged 30–74 years residing in these two municipalities was recruited. The participation rate at baseline was 76 % and information with regard to the CHD outcome was surveyed through 2011 (Fig. 1). Standard questionnaires were used to gain information on previous hospitalisations relevant for our hypothesis, leisure time physical activity, smoking habits, and alcohol habits [10]. In total, 2816 subjects were randomly selected for screening; of these, 50 were excluded because they displayed CHD at baseline and a further 21 individuals were excluded because of unsuccessful endothelin-1 measurements. Thus, 2745 participants were included in the present study.

### Measurements, blood sampling and analyses

The procedures of the investigation have been described previously [11]. Briefly, participants arrived at the study centre in the morning after fasting overnight. Body weight and height were measured with participants in light clothing and without shoes. Body mass index (BMI) was calculated as weight (kg) divided by height squared (m^2^). Waist circumference was measured between the lowest rib margin and the iliac crest and hip circumference at the largest circumference between waist and thighs. Fasting blood samples were drawn and standard resting blood pressure was measured twice 1 min apart with subjects in a supine position with the arm adjusted to the heart level. The mean of two readings was used for further analyses, hypertension was defined in accordance with international expert guidelines (ESC/ESH) [12] and known hypertension was considered as ongoing treatment against high blood pressure. HOMA-IR was estimated to get an assessment of insulin sensitivity [13]. The participants were characterised with respect to diabetes mellitus in accordance with current recommendations from EASD/WHO [14]. Further analyses included apolipoprotein B/apolipoprotein A1 (apoB/apoA1), high-density lipoprotein (HDL), low-density lipoprotein (LDL), testosterone, estradiol, cholesterol, and high sensitivity C-reactive protein (hs-CRP) using standard methods [15–17]. The QuantiGlo Chemiluminescent ELISA for Human endothelin-1 from R&D systems (Minneapolis, MN, USA) was used for the quantitative determination of serum endothelin-1 concentrations (intra-assay precision: CV % 3.4; inter-assay precision: CV % 9.1).

### Ethics

This study was approved by the local ethics committee at the University of Gothenburg (Dnr 199–01) and all participants gave signed informed consent in accordance with the Declaration of Helsinki.

### Follow-up

All participants were followed from the baseline examination until first CHD event or death, or otherwise until December 31, 2011. All events were retrieved by data linkage with the Swedish Cause of Death and Hospital Discharge Registers, which are a reliable validated alternative to revised hospital discharge and death certificates [18, 19]. The outcome considered in this study was CHD defined as non-fatal myocardial infarction (ICD10 code I21), percutaneous coronary intervention (PCI) and/or coronary artery bypass grafting (CABG) or fatal CHD (ICD10 codes I21-I23 and I25) [20].

### Statistical methods

Standard methods were used for descriptive statistics. Linear regression models were used to investigate the association between endothelin-1 and continuous variables while differences between means in continuous variables were analysed by general linear models. Kaplan-Meier survival curves for tertiles of endothelin-1 concentrations were analysed to decide the feasibility of analysis with Cox regressions. After controlling for proportionality, Cox proportional hazard regressions were used to investigate the associations between circulating endothelin-1 levels and CHD. Theoretical multivariate models were used to assess interactions and to estimate the roles of possible confounders as considered by stratification, and by multivariate analyses. The selection of variables for adjustment was based on established risk factors for CHD, as well as apparent correlations with endothelin-1. Differences between men and women were addressed in stratified analyses, as well as in combined analyses using calculations of interaction terms as a statistical tool. All analyses were two-sided, and *p* < 0.05 was considered statistically significant. SPSS Base Systems for Macintosh 18.0 was used for data analyses.

### Availability of data

All the information is stored in databases. More detailed information can be retrieved after contacting the PI of the study (Professor Ulf Lindblad email-ulf.lindblad@allmed.gu.se).

## Results

During the follow-up time (8 ± 1.3 years), 72 events of incident CHD (52 in men and 20 in women) were registered in the Swedish Hospital Discharge Register; of these, 19 events (14 in men and 5 in women) were fatal according to the Swedish Cause of Death Register. Thus, the event rate of incident CHD was 2.6 per 1000 person years (men 4.9 per 1000 person years; women 1.9 per 1000 person years). In total, 88 of the 2745 participants died during the follow-up time.

### Established risk factors and incident CHD

Clinical characteristics for men and women with or without incident CHD are shown in Table 1*.* As expected, age, waist hip ratio (WHR), LDL, apoB/apoA1, systolic and diastolic blood pressure were significantly different between participants with or without incident CHD. Cholesterol, hs-CRP and HOMA-IR only differed in men with or without incident CHD. Hypertension and type 2 diabetes were more common in the group with incident CHD for both men and women. However, BMI, HDL, serum testosterone, serum estradiol, alcohol consumption, current smoking and performing a low level of physical activity were similar in participants irrespective of incident CHD. Treatment with β-blockade against high blood pressure was significantly more frequent in women who developed CHD compared with women without incident CHD while other medications did not significantly differ between the groups. Other indications for treatment with β-blockade were irregular heartbeat (2 men, 10 women); headache or migraine (1 men, 6 women); and tremor (3 men, 1 woman). Participants reporting these indications were all in the group not developing CHD.

**Table 1:** Baseline characteristics of adult residents with and without incident CHD during follow-up in the Vara-Skövde cohort

	Men				Women			
	All *N* = 1350	No CHD *N* = 1298	CHD *N* = 52	*P*-value	All *N* = 1395	No CHD *N* = 1375	CHD *N* = 20	*P*-value
	Mean ± SD				Mean ± SD			
Age (yrs)	47.3±11	46.7 ± 11	61.2±11	<0.001	47.7±12	47.4±12	63.9±11	<0.001
WHR	0.9±0.1	0.9±0.1	1.0±0.1	<0.001	0.8±0.1	0.8±0.1	0.9±0.1	0.004
BMI (kg/m^2^)	26.9±4	26.8±4	27.4±3	0.200	26.8±5	26.8±5	27.8±4	0.661
HOMA-IR	1.6±1.3	1.6±1.3	2.4±1.8	<0.001	1.5±1.6	1.5±1.6	2.1±1.7	0.073
S-LDL (mmol/l)	3.4 ± 0.9	3.4 ± 0.9	3.9 ± 0.9	<0.001	3.2 ± 0.9	3.1 ± 0.9	3.8 ± 1.1	0.001
S-HDL (mmol/l)	1.2 ± 0.3	1.2 ± 0.3	1.2 ± 0.3	0.916	1.4 ± 0.3	1.4 ± 0.3	1.3 ± 0.4	0.164
S-Cholesterol (mmol/l)	5.4 ± 1	5.4 ± 1	5.7 ± 1	0.018	5.3 ± 1.1	5.2 ± 1	5.4 ± 1.2	0.331
ApoB/apoA1	0.6 ± 0.2	0.6 ± 0.2	0.7 ± 0.2	0.002	0.5 ± 0.2	0.6 ± 0.2	0.7 ± 0.2	0.021
S-Triglycerides (mmol/l)	1.5 ± 0.9	1.4 ± 0.9	1.8 ± 1.1	0.018	1.1 ± 0.6	1.1 ± 0.6	1.4 ± 0.9	0.003
Hs-CRP (mg/l)	2.4 ± 5.8	2.2 ± 4.2	6.1 ± 21	<0.001	2.8 ± 4.6	2.8 ± 4.6	3.2 ± 3.9	0.675
Systolic blood pressure (mmHg)	124 ± 16	123 ± 15	138 ± 19	<0.001	119 ± 18	119 ± 18	143 ± 20	<0.001
Diastolic blood pressure (mmHg)	72 ± 10	72 ± 10	78 ± 12	<0.001	69 ± 10	68 ± 10	77 ± 10	<0.001
Alcohol consumption (g/week)	62 ± 108	62 ± 108	55 ± 91	0.666	31 ± 43	31 ± 44	20 ± 36	0.278
S-Testosterone (nmol/l)	14 ± 4.3	14 ± 4.3	13 ± 4.7	0.147	1.3 ± 1.3	1.3 ± 1.3	1.1 ± 0.5	0.524
S-Estradiol (nmol/l)	125 ± 37	125 ± 37	126 ± 40	0.875	304±381	305 ± 383	221 ± 50	0.314
	*N* (%)				*N* (%)			
Hypertension	183 (14)	165 (13)	22 (42)	<0.001	204 (14)	192 (14)	12 (57)	<0.001
Type 2 diabetes	78 (5.7)	66 (5)	12 (23)	<0.001	65 (4.6)	61 (4.4)	4 (19)	0.004
Daily smoking	214 (16)	201 (15)	13 (25)	0.068	288 (21)	282 (20)	6 (29)	0.364
Low physical activity	521 (38)	500 (40)	21 (45)	0.742	384 (27)	382 (29)	2 (10)	0.088
Medication								
ARB	14 (1.0)	13 (1.0)	1 (1.9)	0.886	13 (0.9)	13 (1.0)	0 (0)	0.999
ASA	31 (2.2)	27 (2.1)	4 (7.7)	0.991	34 (2.4)	31 (2.2)	3 (14)	0.426
Statin	25 (1.8)	22 (1.7)	3 (5.8)	0.673	42 (2.9)	38 (2.7)	4 (19)	0.247
β-Blocker	64 (4.7)	58 (4.4)	6 (11.5)	0.830	89 (6.3)	80 (5.8)	9 (43)	0.007
ACE inhibitor	24 (1.8)	21 (1.6)	3 (5.8)	0.716	29 (2)	26 (1.9)	3 (14)	0.166
Metformin	14 (1.0)	13 (1.0)	1 (1.9)	0.992	19 (1.3)	18 (1.3)	1 (4.8)	0.988
HRT	-	-	-	-	247 (17)	238 (17)	9 (43)	0.434

All means are adjusted

All means are adjusted for differences in age. CHD: coronary heart disease; WHR: waist hip ratio (missing 10/2745); BMI: body mass index (missing 3/2745); HOMA-IR: homeostatic model assessment of insulin resistance (missing 39/2745); LDL: low-density lipoprotein (missing 2/2745); HDL: high-density lipoprotein (missing 2/2745); ApoB/apoA1: apolipoprotein B/apolipoprotein A1 (missing 4/2745), hs-CRP: high sensitivity c-reactive protein (missing 2/2745); testosterone (missing 135/2745); estradiol (missing 30/2745); type 2 diabetes (missing 2/2745); physical activity (missing 91/2745); ARB: angiotensin II receptor blockers; ASA: acetyl-salicylic acid; ACE inhibitors: angiotensin converting enzyme inhibitor; HRT: hormone replacement therapy. 12 g alcohol is equivalent to approximately 1 glass of wine (12–15 cl) or 1 small beer (33 cl) (missing 126/2745). Differences in continuous variables were investigated using general linear models. Differences in dichotomous variables were analysed using logistic regression analyses

### Associations between clinical variables and circulating endothelin-1 levels at baseline

**Fig 2:**
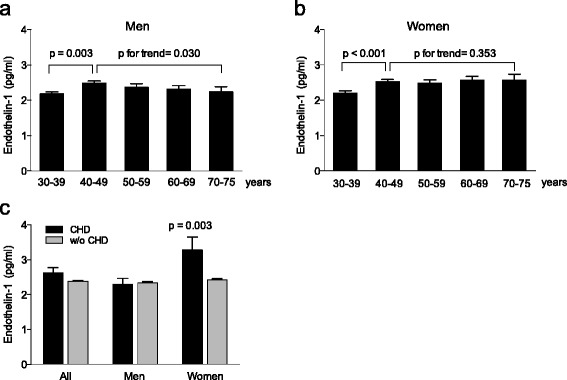
Circulating concentrations of endothelin-1 at baseline separated by age in the Vara-Skövde Cohort in (**a**) men and (**b**) women. **c** Endothelin-1 levels at baseline comparing participants with incident CHD (men *n* = 52, women *n* = 20) and participants without coronary heart disease (CHD) (men *n* = 1307, women *n* = 1386) at the follow-up. General linear model was used in statistical analyses, mean ± SEM

Circulating endothelin-1 levels were lowest at age 30–39 years and increased significantly at age 40–49 years for both men and women; however, endothelin-1 levels then declined in a stepwise fashion in men (*p* = 0.003) (Fig. 2a) but remained constant in women (Fig. 2b). Circulating endothelin-1 levels were similar in participants with or without hypertension (women: 2.4 pg/ml, SE: 0.04 vs 2.5 pg/ml, SE: 0.10, *p* = 0.801; men: 2.3 pg/mL, SE: 0.04 vs 2.3 pg/ml, SE: 0.09, *p* = 0.434). Linear regression analyses were used to explore the association of circulating endothelin-1 levels with other clinical variables (Table 2). Circulating endothelin-1 levels were associated with both insulin resistance (HOMA-IR) and apoB/apoA1 ratio in men whereas no such associations were observed in women (Table 2). Furthermore, circulating testosterone levels were associated with circulating endothelin-1 levels in men (Table 2). Finally, HDL cholesterol showed a positive association with circulating endothelin-1 levels in women.

**Table 2:** Associations between circulating endothelin-1 levels and selected variables at baseline in men and women from the Vara-Skövde cohort

	Men (*n* = 1350)		Women (*n* = 1395)	
	β	*P*	β	*P*
Age	0.029	0.290	0.090	0.001
BMI	0.025	0.343	0.015	0.563
WHR	0.023	0.237	0.001	0.999
HOMA-IR	0.066	0.016	0.031	0.262
Systolic BP	0.026	0.392	−0.029	0.386
Diastolic BP	0.012	0.663	−0.010	0.854
S-HDL	0.010	0.695	0.065	0.015
S-LDL	0.030	0.154	0.040	0.354
ApoB/apoA1	−0.064	0.018	0.001	0.999
Triglycerides	0.029	0.289	−0.018	0.697
CRP	−0.020	0.437	0.015	0.542
Glucose	0.026	0.352	−0.014	0.618
LTPA	−0.017	0.541	0.022	0.432
Smoking habits	0.007	0.776	0.018	0.524
Testosterone	−0.055	0.048	0.006	0.816
Estradiol	0.001	0.990	0.005	0.861

Age-adjusted linear regression analysis to investigate the association between concentrations of endothelin-1 and factors related to coronary heart disease

*HOMA-IR* homeostatic model assessment of insulin resistance, *BMI* body mass index, *WHR* waist/hip ratio, *BP* blood pressure, *HDL* high-density lipoprotein, *LDL* low-density lipoprotein, *ApoB/apoA1* apolipoprotein B/apolipoprotein A1, *CRP* C-reactive protein, *LTPA* leisure time physical activity

### Circulating endothelin-1 levels and incident CHD

Circulating levels of endothelin-1 at baseline were not different between men with or without incident CHD in the follow-up period. By contrast, circulating endothelin-1 levels at baseline in women with CHD at follow-up were significantly higher than in women without incident CHD (Fig. 2c). In women, the age at the CHD event ranged between 44 and 82 years and 19 out of 20 women were postmenopausal (≥55 years old) when they got their event.

**Fig 3:**
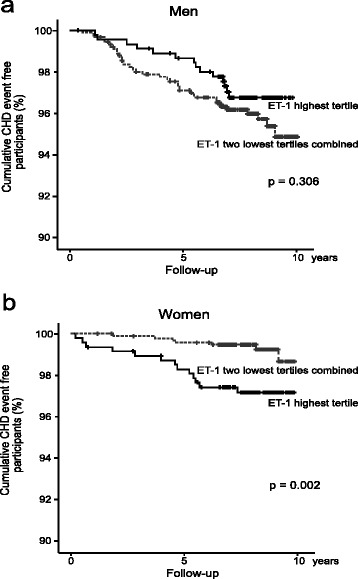
Kaplan-Meier survival plots comparing time to the first coronary heart disease (CHD) event between participants with the highest tertile of endothelin-1 (ET-1) and the remaining population in (**a**) men (highest tertile *n* = 453, two lower tertiles combined *n* = 906) and (**b**) women (highest tertile *n* = 469, two lower tertiles combined *n* = 938). + = Censored

Women showed a strong association between higher circulating levels of endothelin-1 and CHD after adjustment for age (HR = 1.51 CI: 1.1–2.1 *p* = 0.015), but this association was not significant in men (HR = 0.98, CI 0.8–1.2, *p* = 0.854) (Table 3). The association between endothelin-1 levels and CHD in women also remained significant after adjusting for age, HOMA-IR, apoB/apoA1 and smoking habits (HR = 1.51 CI 1.1–2.1 *p* = 0.024) (Table 3). Further, in women the association between endothelin-1 levels and CHD remained significant after adjustment for age in combination with WHR, BMI, LDL-cholesterol, HDL-cholesterol, hypertension, CRP, triglycerides or medications (data not shown). Kaplan-Meier survival curves showed no significant difference in CHD event-free time in men depending on their circulating endothelin-1 concentrations (Fig. 3a). However, the curves showed significantly shorter CHD event-free time for women in the highest tertile of circulating endothelin-1 concentrations when compared with women in the two lower tertiles (*p* = 0.002) (Fig. 3b). Finally, an interaction-term assessing the relationship between endothelin-1 and sex was significant (*p* = 0.011).

**Table 3:** Predictive value of baseline circulating endothelin-1 levels on CHD in men and women in the Vara-Skövde cohort

Men (*n* = 1350)			Women (*n* = 1395)		
HR	CI	*p*	HR	CI	*p*
Crude					
0.98	0.8–1.2	0.818	1.64	1.2–2.3	0.003
Adjusted for age					
0.98	0.8–1.2	0.854	1.51	1.1–2.1	0.015
Adjusted for age, HOMA-IR, apoB/apoA1 and smoking					
0.88	0.7–1.1	0.272	1.53	1.1–2.1	0.024

Cox-regression analysing the hazard ratio for CHD during follow-up time for changes of endothelin-1 concentrations with 1 pg/ml

*HR* hazard ratio, *CI* confidence interval, *ApoB/apoA1* apolipoprotein B/apolipoprotein A1, *HOMA-IR* homeostatic model assessment of insulin resistance

## Discussion

In this large observational Swedish study, increased circulating endothelin-1 levels were associated with incident CHD in women but not in men. This was independent of other risk factors for CHD and circulating levels of estradiol. This suggests that endothelin-1 may be an important independent predictor of CHD risk in woman. To our knowledge, this is the first time a study prospectively shows the predictability of circulating endothelin-1 levels for incident CHD in women.

Our main observation suggests sex-differences in the association between endothelin-1 and the outcome in our population, and this is also supported by a significant interaction term. However, no association between circulating levels of estradiol and endothelin-1 was found. Earlier epidemiological and experimental studies have shown that exogenous estradiol decreases endothelin-1 levels whereas testosterone levels have been shown to correlate positively with circulating endothelin-1 levels [21, 22]. Similarly, women receiving hormone replacement therapy in the Rancho Bernardo cohort displayed lower circulating endothelin-1 levels and administration of estrogen promotes vasodilation in humans and in experimental animals [7, 23, 24]. In addition, the Rancho Bernardo study showed an association between CHD and endothelin-1 in older men [7]. The current study did not confirm this finding, likely because of the younger age of the participants and a low number of CHD events in the population (2.6 % in men, 1.4 % in women). Interestingly, reduced circulating endothelin-1 levels were observed in men over 50 years old. This observation needs to be confirmed in larger studies.

As expected, a number of baseline characteristics differed between participants who developed CHD and those who remained healthy during the follow-up, and some of these traits also showed associations with circulating endothelin-1 levels at baseline. Interestingly, these variables differed between men and women. In agreement with earlier results, testosterone levels correlated with endothelin-1 concentrations in men as discussed above [21] and HOMA-IR also correlated with endothelin-1 levels in men [25]. Moreover, apoB/apoA1 was negatively associated with endothelin-1 levels in men, which was surprising, but currently little is known about this correlation. The associations between age and endothelin-1 levels in women confirm the results from previous investigations [26]. However, the positive correlation between endothelin-1 and HDL in women was unexpected and suggests that endothelin-1 may be beneficial for HDL metabolism. To our knowledge, this association has not been observed previously, and should be investigated further. The main observation that endothelin-1 predicts CHD in women was not affected when adjusting for clinical traits that differed between the groups. Furthermore, an increase in circulating endothelin-1 concentrations in participants diagnosed with hypertension was not observed in this study, which is in line with previous observations [27], but in contrast with other observations [28]. Different expression of endothelin-1 receptors in vascular territories of the human body [29] may underlie the link between endothelin-1 and coronary outcome and the lack of an association between endothelin-1 and systemic blood pressure.

Further, 43 % of women with incident CHD and 5.8 % of women who remained healthy during follow-up were treated with β-blockade for hypertension at baseline. Although earlier studies report that β-blockade does not affect calf blood flow in patients with chronic stable intermittent claudication [30], a small study suggested that β-blockade may be related to worsening of peripheral blood flow [31]; however, very little has been published on the effect of β-blockade on the microcirculation. We suggest that this possibility should be elucidated in future studies.

### Strengths and limitations

Some strengths and weaknesses of this survey need to be mentioned. Advantages of this study include the high participation rate, the unselected study population and the prospective design allowing us to investigate the direction of the observed associations. However, a weakness is the low number of CHD events and the results need to be confirmed in larger population samples. Thus, the use of multivariate Cox regression analysis in subgroups conveys risks to over interpret the associations, but the predefined hypothesis in this study should overcome this problem [32]. Importantly, inclusion of participants aged 30–74 years allowed us to prospectively investigate the impact of circulating endothelin-1 levels on incident CHD in a population less confounded by comorbidity. Another limitation of this study is the lack of information regarding menopause in women. Given the strong interaction between endothelin-1 and NO in the endothelium, models including determinants of NO-production should strengthen the results and might be object of further research.

## Conclusion

In conclusion, the results of this prospective study indicate that endothelin-1 may predict development of CHD in women. Trials investigating the effect of endothelin-1 receptor antagonists or other means to inhibit endothelin-1 for prevention of CHD in high-risk women are still lacking and could better address the question of causality.
